# Asystole following positive pressure insufflation of right pleural cavity: a case report

**DOI:** 10.1186/1752-1947-5-257

**Published:** 2011-06-30

**Authors:** Kari M Forde-Thielen, Mojca R Konia

**Affiliations:** 1 Department of Anesthesiology, University of Minnesota, Box 294, B515 Mayo Memorial Building, 420 Delaware Street, SE, Minneapolis, MN 55455, USA

## Abstract

**Introduction:**

Adverse hemodynamic effects with severe bradycardia have been previously reported during positive pressure insufflation of the right thoracic cavity in humans. To the best of our knowledge, this is the first report of asystole during thoracoscopic surgery with positive pressure insufflation.

**Case presentation:**

A 63-year-old Caucasian woman developed asystole at the onset of positive pressure insufflation of her right hemithorax during a thoracoscopic single-lung ventilation procedure. Immediate deflation of pleural cavity, intravenous glycopyrrolate and atropine administration returned her heart rhythm to normal sinus rhythm. The surgery proceeded in the absence of positive pressure insufflation without any further complications.

**Conclusions:**

We discuss the proposed mechanisms of hemodynamic instability with positive pressure thoracic insufflation, and anesthetic and insufflation techniques that decrease the likelihood of adverse hemodynamic events.

## Introduction

Hemodynamic consequences of pleural cavity positive pressure insufflation during thoracoscopic procedures have been described in the literature. While some of these reports show minimal clinically significant effects, others show serious hemodynamic consequences. One potentially severe complication, asystole, has not previously been reported. We present the case of a female patient who underwent right thoracoscopic mediastinal lymph node dissection with one-lung ventilation and developed hypotension and asystole at the commencement of positive pressure insufflation of her right hemithorax. We discuss the proposed mechanisms for the development of hemodynamic changes and asystole as well as ways in which the safety of thoracoscopic surgery can be improved.

## Case presentation

A 63-year-old Caucasian woman with subcarinal and left hilar lymphadenopathy presented to our hospital for a right thoracoscopic mediastinal lymph node dissection. Our patient had a history of endometrial adenocarcinoma, and lymphadenopathy was noted on a follow-up computed tomography scan. At the time of the initial presentation, three years ago, the carcinoma was treated surgically. The post-surgical course at that time was complicated with deep vein thrombosis and pulmonary embolism, which were treated with anticoagulation therapy and the placement of an inferior vena cava filter. Our patient denied any residual shortness of breath or limitation of activity. She was participating in water aerobics and was able to walk up a flight of stairs easily without shortness of breath. Her history was also significant for gastroesophageal reflux disease and dyslipidemia. Our patient's medications included ranitidine and simvastatin. She did not report any allergies. A physical exam was unremarkable except for obesity (weight 119 kg; height 165 cm).

Standard American Society of Anesthesiologists (ASA) monitors were placed. After a rapid sequence induction (fentanyl 1 μg/kg, lidocaine 1 mg/kg, propofol 2 mg/kg, succinylcholine 1.5 mg/kg) our patient was intubated with a 39 French left-sided double lumen endotracheal tube. Anesthesia was maintained with desflurane 5-6%, fentanyl (total intraoperative dose 300 μg), vecuronium 4 mg and fraction of inspired oxygen (FiO2) at 1.0. Our patient was turned to the left lateral position and the correct position of the endotracheal tube was confirmed with fiberoptic bronchoscopy. Left sided one-lung ventilation was initiated (tidal volume 300cc, respiratory rate 16/min, positive-pressure respiration (PEEP) 4, FiO2 1.0). Incisions were made soon after initiation of one-lung ventilation, and ports were inserted. Vitals signs at the time of incision included saturated oxygen 99-100%, noninvasive blood pressure 150/75 mmHg and pulse 80/min. Her right hemithorax was insufflated with carbon dioxide to a pressure of 10 mmHg at a rate of 25 L/min. At this point in the procedure we noted changes in our patient's hemodynamic status. Her blood pressure dropped to 112/63 and her heart rate precipitously dropped to 35/min for a few seconds. This was followed by asystole. The surgeon was notified, insufflation was stopped and glycopyrrolate 0.4 mg and atropine 0.4 mg were administered. Within 10 seconds of pleural deflation our patient resumed a normal sinus rhythm. Her next blood pressure reading was 125/75. In the absence of further rhythm or hemodynamic abnormalities the surgery was continued. The pleural cavity was slowly insufflated to a pressure of 8 mmHg and the case progressed without any further complications. Our patient had an uncomplicated post-operative course.

## Discussion

Insufflation of the pleural cavity with carbon dioxide is believed by some to facilitate lung deflation during one-lung ventilation and improve surgical exposure. Insufflation also enlarges the intrapleural space by pushing the mediastinum away from the operative field [1]. The technique is often used in thoracoscopic harvesting of the internal mammary artery. The practice of insufflation has further been used successfully in collapsing emphysematous lung [1]. It has, however, been suggested that insufflation is unnecessary for adequate exposure in the majority of thoracoscopic surgeries and may increase the risk of hemodynamic compromise [2-4]. It has also been suggested that opening a port to air is safer than and just as effective as insufflations [5].

The initial reports of significant hemodynamic compromise during positive pressure insufflation of the pleural cavity came from pig studies [6]. The anatomy and physiology of a pig, however, is significantly different from that of a human [7]. Based on human studies, most authors agree that insufflation of the hemithorax up to a pressure of 10 mmHg with one-lung ventilation causes some hemodynamic change. Right-sided insufflation causes more pronounced hemodynamic effect compared to left-sided insufflation because the right heart is a low-pressure system [1,5,6]. Human studies have demonstrated that positive pressure insufflation of the right or left pleural cavity increases central venous pressure (CVP) by 4-10 mmHg [1,7,8] and pulmonary artery (PA) pressure by 2-7 mmHg [1,7], which ultimately results in a decrease in preload. The effects of positive pressure insufflation of the pleural cavity have less significant effects on systemic pressures and cardiac function [3,4,7-9].

There are several theories that explain the mechanisms of hemodynamic changes. Positive pressure insufflation of the thoracic cavity creates physiologic effects similar to a tension pneumothorax [6]. This effect can explain the increase in CVP and decrease in venous return. More pronounced effects in certain reports could be explained by higher than intended intrathoracic pressures caused by incomplete lung collapse at initiation of pleural cavity insufflation. In this situation the pleural pressure may increase enough to close off the small airways. Respiratory gases get trapped in the lung and further insufflation induces a rapid increase in intrathoracic pressure instead of collapsing the lung. The high intrathoracic pressure further reduces venous return and lowers the cardiac output [5].

Increases in mean PA pressure and pulmonary capillary wedge pressure may be caused by positive intrapleural pressure together with hypoxic pulmonary vasoconstriction in the collapsed pulmonary parenchyma [1,7].

There are no prior reports of asystole on commencement of pleural insufflation during one-lung ventilation in the human literature. However, Harris *et al. *reported a case of severe bradycardia following hypotension during right-sided thoracoscopic dorsal sympathectomy with insufflation up to a pressure of 15 mmHg [5]. Our case began with mild hypotension and bradycardia which progressed to asystole, suggesting that these hemodynamic changes are all part of a spectrum of derangements caused by the same inciting mechanism.

The incidence and mechanisms of bradycardia and asystole are poorly defined. Besides the hemodynamic effects described above, increased intrathoracic pressure could cause direct vagal stimulation and thereby induce bradycardia or asystole. Some authors have proposed a Bezold-Jarisch reflex as an underlying mechanism [5]. The Bezold-Jarisch reflex occurs when a patient with an intact autonomic nervous system experiences hypotension. This is sensed by baroreceptors in the aortic arch and carotid sinuses, which compensate with greatly increased cardiac contractility. The cardiac baroreceptors then sense this high intramural tension and induce significant parasympathetic discharge resulting in bradycardia or asystole.

To explain asystole in our patient, we turn to the sequence of events to help elucidate the pathophysiology. After positioning of our patient and preparation of the surgical field we initiated one-lung ventilation. Ports were immediately inserted and positive pressure insufflation of her right pleural cavity with high flows (25 L/min) to a pressure of 10 mmHg was started. Full deflation of the lung was not carefully assessed prior to positive pressure insufflation. As demonstrated by Jacobs *et al*., the actual pressure in an insufflated cavity may briefly be much higher than the insufflator pressure presetting [10]. Temporary deviations of up to 78.8% from the displayed pressure were observed during their study in laparoscopic procedures. We propose that the lung of our patient was not fully deflated at initiation of insufflation. The intrathoracic pressure increased instantaneously with high flows of insufflation and most likely exceeded 10 mmHg, at least temporarily. This caused a pronounced mediastinal shift, an immediate drop in venous return, with little time for the sympathetic nervous system to slowly compensate, and either vagal stimulation or activation of the Bezold-Jarisch reflex leading to asystole.

## Conclusion

In the literature it is suggested that insufflation flows should be kept at 2-3 L/min [1,5,7]. In the authors' experience the insufflation flows vary and high flows are often utilized, which may not promote patient safety. Also, with rapid insufflation using high flows the insufflator may overshoot and increase intrathoracic pressure over the safe limit. We therefore propose that, to prevent events like this from occurring, insufflation pressures should be kept below 10 mmHg [5,9]. We suggest giving enough time for full deflation of the lung. It would be prudent to initiate one-lung ventilation as soon as possible and use 100% oxygen to accelerate lung deflation [11]. It has also previously been recommended that the lung should be exposed to ambient pressure for 60 seconds before the initiation of insufflations [5]. This will allow for hypoxic pulmonary vasoconstriction to limit shunting and for the sympathetic response to compensate for decreased venous return. Insufflation should be used primarily in patients with unsatisfactory surgical exposure following passive lung deflation.

## Consent

Written informed consent was obtained from the patient for publication of this case report. A copy of the written consent is available for review by the Editor-in-Chief of this journal.

## Competing interests

The authors declare that they have no competing interests.

## Authors' contributions

Both authors participated in the clinical care of the patient and participated in the collection and review of the literature and writing of the case report. Both authors have read and approved the final manuscript.
